# High-Frequency Stimulation of Excitable Cells and Networks

**DOI:** 10.1371/journal.pone.0081402

**Published:** 2013-11-20

**Authors:** Seth H. Weinberg

**Affiliations:** Department of Applied Science, The College of William and Mary, Williamsburg, Virginia, United States of America; University of Maribor, Slovenia

## Abstract

High-frequency (HF) stimulation has been shown to block conduction in excitable cells including neurons and cardiac myocytes. However, the precise mechanisms underlying conduction block are unclear. Using a multi-scale method, the influence of HF stimulation is investigated in the simplified FitzhHugh-Nagumo and biophysically-detailed Hodgkin-Huxley models. In both models, HF stimulation alters the amplitude and frequency of repetitive firing in response to a constant applied current and increases the threshold to evoke a single action potential in response to a brief applied current pulse. Further, the excitable cells cannot evoke a single action potential or fire repetitively above critical values for the HF stimulation amplitude. Analytical expressions for the critical values and thresholds are determined in the FitzHugh-Nagumo model. In the Hodgkin-Huxley model, it is shown that HF stimulation alters the dynamics of ionic current gating, shifting the steady-state activation, inactivation, and time constant curves, suggesting several possible mechanisms for conduction block. Finally, we demonstrate that HF stimulation of a network of neurons reduces the electrical activity firing rate, increases network synchronization, and for a sufficiently large HF stimulation, leads to complete electrical quiescence. In this study, we demonstrate a novel approach to investigate HF stimulation in biophysically-detailed ionic models of excitable cells, demonstrate possible mechanisms for HF stimulation conduction block in neurons, and provide insight into the influence of HF stimulation on neural networks.

## Introduction

Electrical signaling is fundamental to the physiological function of excitable cells such as neurons and cardiac myocytes. Irregular electrical patterns in the brain and heart can lead to life-threatening conditions including epileptic seizures and ventricular fibrillation. External stimulation can terminate these irregular rhythms [1], [2]; however large strength stimuli are often associated with detrimental effects such as pain [3] and impaired cardiac function following defibrillation [4].

In the 1960s, it was shown that kilohertz-range high frequency (HF) sinusoidal stimulation could reversibly block conduction in neurons [5]. The use of 1–40 kHz HF-induced neural conduction block has recently been exploited in clinical studies for diagnostic and therapeutic purposes, improving bladder function [6], [7] and mitigating pain associated with peripheral nerve activity [8]–[10]. Despite the clinical usage of HF stimulation treatment, the mechanisms underlying therapeutic success in these physiological and pathological settings are unclear. Simulation studies in neurons have suggested two mechanisms: reduced sodium channel availability due to transmembrane potential depolarization and persistent activation of potassium channels [8]–[14]. However, the relative significance of the two mechanisms varies with the properties of the neuron, as well as the specific species and model. Further, in simulation studies, the transmembrane potential, ionic currents, and channel gating variables oscillate on the fast time scale of the HF stimulus, varying throughout the HF stimulation period, such that distinguishing the precise influence of the HF stimulus is difficult.

Alternatively, one can apply a multi-scale method, separating the fast time scale dynamics—due to the HF stimulus—and the slow dynamics of the excitable cell, and derive an *averaged* model, which accounts for the HF stimulus but does not contain a high-frequency term [15]. Using this type of approach, several studies have analyzed the influence of a HF stimulus in the simple FitzHugh-Nagumo (FHN) model [16]. Cubero and colleagues demonstrated that the model cell cannot repetitively fire when the HF stimulus amplitude-frequency ratio is above a critical value [17]. Ratas and Pyragas showed that this ratio also influenced conduction speed in a nerve axon and above a critical value led to conduction block [18], [19]. The FHN model is minimalistic, reproducing many important aspects of cellular excitability [20], and ideal for analysis, as the model only contains two variables, permitting the use of standard nonlinear dynamics techniques such as phase-plane analysis. However, in general biophysically-detailed models of excitable cells are more complex than represented by the simple two-dimensional FHN model.

In this study, we first illustrate the multi-scale method to derive the averaged FHN (AFHN) model equations and use phase-plane analysis to determine critical HF stimulus thresholds above which the model cannot exhibit repetitive firing or elicit a single action potential. We then extend this approach to simulate the dynamics of the classical Hodgkin-Huxley (HH) neuron model [21] and illustrate similarities and differences between the AFHN and averaged Hodgkin-Huxley (AHH) model. Further, we demonstrate that HF stimulation alters the ionic current activation and inactivation dynamics, illustrating possible mechanisms for conduction block in a single neuron. Finally, we simulate HF stimulation in a network of neurons and demonstrate that HF stimulation alters network synchrony and, above a critical stimulation strength, terminates persistent network activity, suggesting implications for clinical therapy.

## Results

### Averaged Fitzhugh-Nagumo model

We begin by deriving and analyzing a simplified model of spiking which accounts for the influence of HF stimulation. We consider the FitzHugh-Nagumo (FHN) model [16] with the addition of a constant current 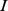 and a time-varying HF stimulus
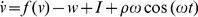
(1a)




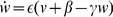
(1b)


where 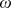 is the frequency of the HF stimulation, 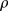 is the HF amplitude-frequency ratio,
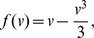



and the dot indicates differentiation with respect to time 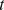. The HF stimulation term is defined in terms of 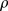 and 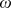 with the foresight that the influence of the HF stimulation depends on 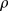, not specifically the amplitude 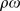. The FHN model is a simplified model that reproduces many important properties and dynamics of excitable cells. The simplicity of the model permits a geometric illustration—through phase plane analysis—of many important biophysical phenomena such as repetitive spiking and depolarization block. In the model, 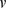 is the dimensionless transmembrane potential, and 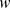 represents the degree of refractoriness. Throughout the paper, we fix 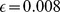, 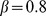, and 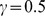, such that in the absence of any external stimuli, the neuron is excitable.

If the period of the fast HF stimulus is much smaller than all characteristic times of the FHN model, according to the method of averaging [15], an approximation to the slow system can be obtained by averaging over the period of the HF stimulus. As shown in the Methods, the variables of the averaged Fitzhugh-Nagumo model (AFHN), 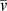 and 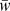, are governed by the following system of equations:
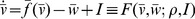
(2a)

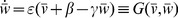
(2b)


where
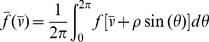


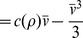



and
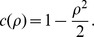



**Figure 1:**
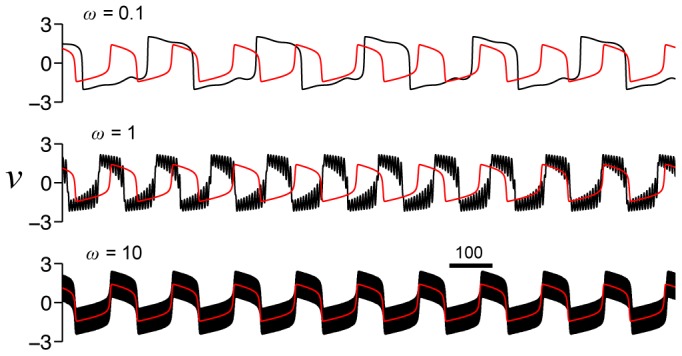
Validation of the AFHN model. Simulated 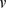 traces from the FHN (black) and AFHN (red) models for varying HF stimulus frequency 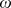. Parameters: Radial frequency 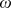 is identified in each panel, 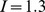, 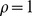.

The AFHN model is very similar to the FHN model, with the only difference being the modification of the cubic function 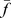 that influences the dynamics of 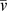. In the absence of HF stimulation, i.e., 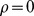, the two models are identical. In the following sections, we investigate how the HF stimulus parameter 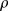 influences the properties of repetitive action potential firing. In Figure 1, we plot 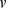 from simulations of the FHN model (black) for various values of the HF stimulation frequency 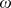 and compare with 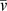 from a simulation of the AFHN neuron model (red), for fixed values for 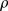 and 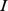. In general, as 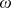 increases, 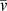 from AFHN model becomes a better approximation of the average value of 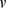 from the FHN model simulation, validating our formulation.

**Repetitive firing in the AFHN model**. In the parameter region considered in this study, the cell is excitable, that is, in the absence of an external stimulus, the cell is at rest, and the addition of a stimulus can induce a single or multiple action potentials. In this study, we will consider two types of applied current stimuli: a constant applied current and a brief applied current pulse, in addition to the HF stimulation.

**Figure 2:**
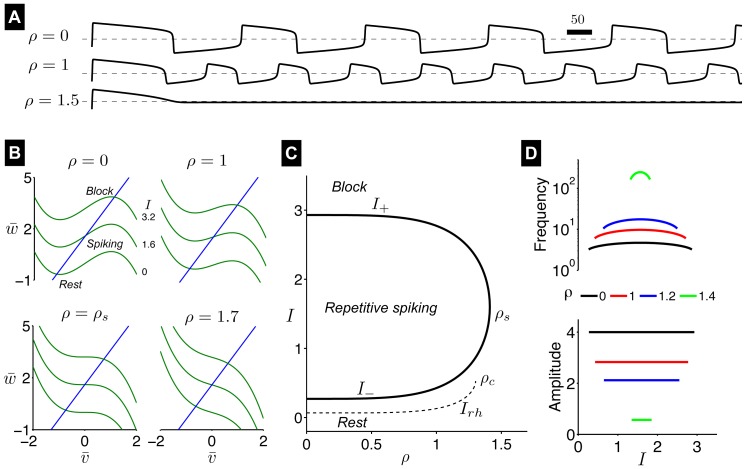
Repetitive firing in the AFHN model. (A) Simulated 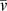 traces for 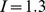 and different values for 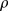. The dashed lines indicate 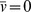. (B) Phase-plane portrait for variable 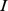 and 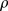. In each panel, the 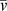-nullcline (green) is shown for 3 values of 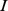. The 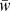-nullcline (blue) is independent of 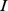 and 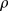. (C) 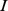-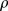 parameter space, denoting regions of rest, repetitive firing, and block. The limit cycle lower and upper limits (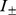, Eq. 6 ) and rheobase (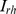, Eq. 9 ) as functions of 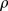. (D) Frequency and amplitude of action potentials, as functions of 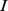 and 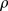.

We first consider the case of a constant applied current 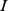. In Figure 2A, we plot 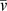 for a constant current 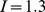 and various values of 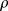. For no HF stimulus (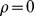), the cell fires repetitively. Increasing the amplitude of the HF stimulation parameter 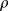 decreases the action potential amplitude and increases the firing frequency. Consistent with previous studies [17], increasing 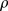 further results in cessation of repetitive firing, following a single action potential at the stimulus onset. Conditions for cessation of firing are derived as follows.

**Figure 3:**
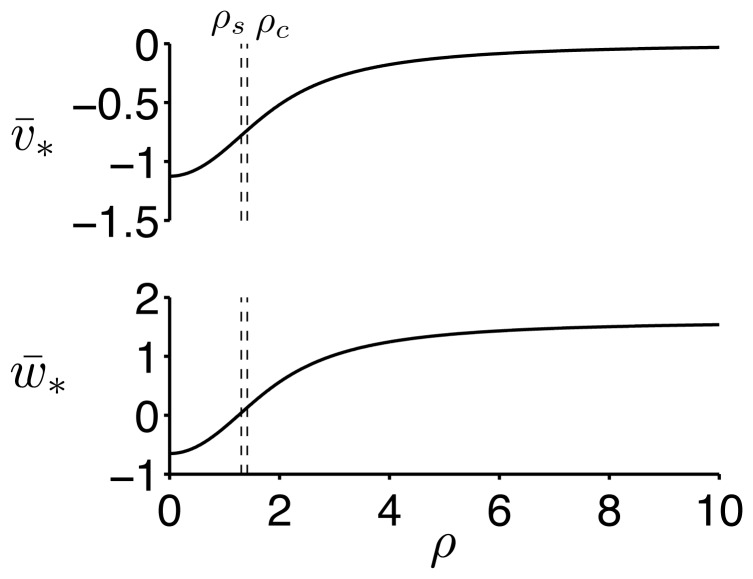
Steady-state of the AFHN model. The steady-state transmembrane potential 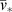 and degree of refractoriness 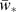 are shown as functions of the HF stimulation parameter 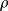. Critical values of 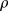 for repetitive firing 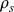 and for evoking a single action potential following a brief applied current pulse 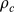 are identified. See text for description of critical values.

For the parameters chosen, the AFHN model has a single steady-state 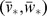, which satisfies the implicit expression
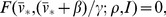
(3a)

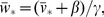
(3b)


and shown in Figure 3. As 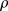 increases, the resting potential 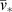 becomes more depolarized and approaches 0 for large 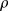. The degree of refractoriness also increases as 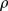 increases, such that 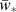 approaches 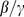 for large 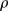.

Using standard techniques from linear stability analysis [22], the stability of the steady-state 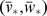 can be determined by linearizing around 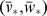, and evaluating the matrix of partial derivatives, the Jacobian 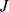, at the steady-state,



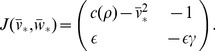
(4)


When the steady-state becomes unstable, specifically the real part of the eigenvalues of 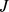, 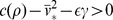, a stable limit cycle emerges, which can be interpreted biophysically as repetitive action potential firing. The critical parameter value at which the limit cycle emerges is known as a *Hopf bifurcation*. Many previous studies have shown that in the FHN model (i.e., 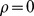), as the applied current 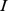 increases, there are two critical values for 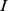, 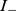 and 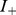, which correspond to the onset and offset of the stable limit cycle, respectively [23]–[25]. Below 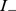, the steady-state is stable corresponding to the cell at rest, between 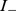 and 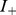 the steady-state is unstable and the cell repetitively fires, and above 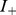, the steady-state is stable again and the cell is in depolarization block [23].

In Figure 2B, we plot the nullclines of the AFHN model for several values of 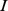 and 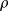. The 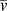-nullcline (green)—given by the set of all points 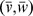 such that 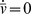
—is a cubic function of 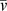, while the 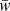-nullcine (blue)—similarly defined as the set of all points 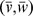 such that 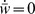
—is linear, and the nullclines intersection denotes the location of the steady-state. For a given value of 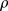, increasing 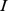 shifts the 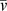-nullcline upwards, while the 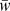-nullcline is independent of both 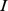 and 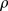.

If 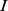 is such that the steady-state is located on the middle branch of the 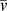-nullcline, and if 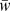 is sufficiently slow compared to 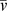, that is, 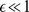, then it can be shown that the steady-state is unstable, and a stable limit cycle exists [23]. From a geometric illustration, we can anticipate a critical value of 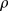, 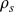, above which a stable limit cycle and repetitive firing cannot exist, consistent with Figure 2A (bottom panel). As 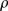 increases, the slope of the middle branch of the 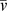-nullcline decreases, and the “knees” of the nullcline move towards the steady-state 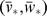. When the slope at 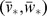 equals 0, the middle branch of the nullcline no longer exists and, therefore regardless of 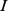, a stable limit cycle also does not exist. Using the slope of the 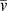-nullcline alone as a criterion for the critical value of 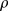, 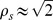.

From linear stability analysis, we can more precisely determine the necessary condition for a limit cycle, 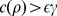, such that 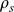 is given by



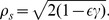
(5)


For all values of 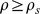 the steady-state is always stable, regardless of 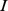, as previously shown by [17]. Further, for 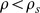, the critical stimulus upper and lower limits, 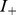 and 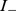, respectively, are given by



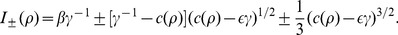
(6)


The 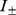 curves separate the regions of rest, repetitive firing, and depolarization block in the 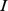-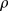 parameter space and coalesce when 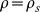 at a double Hopf bifurcation (Figure 2C). For the parameters used in this study, 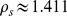.

In the regime for repetitive firing, we derive an approximation for the action potential frequency and amplitude in the AFHN model (see Methods). For a given value of 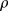, the frequency first increases then decreases as 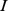 increases (Figure 2D), while the amplitude is constant, consistent with a relaxation oscillator. Increasing 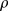 increases frequency and decreases the action potential amplitude, consistent with Figure 2A.

**Excitability in the AFHN model**. We next consider the excitability of the AFHN model following a brief applied current, in the presence of HF stimulation, by determining the *strength-duration curve*, the relationship between the duration 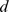 of an applied current pulse and the minimum amplitude 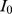 such that an action potential fires [26].

**Figure 4:**
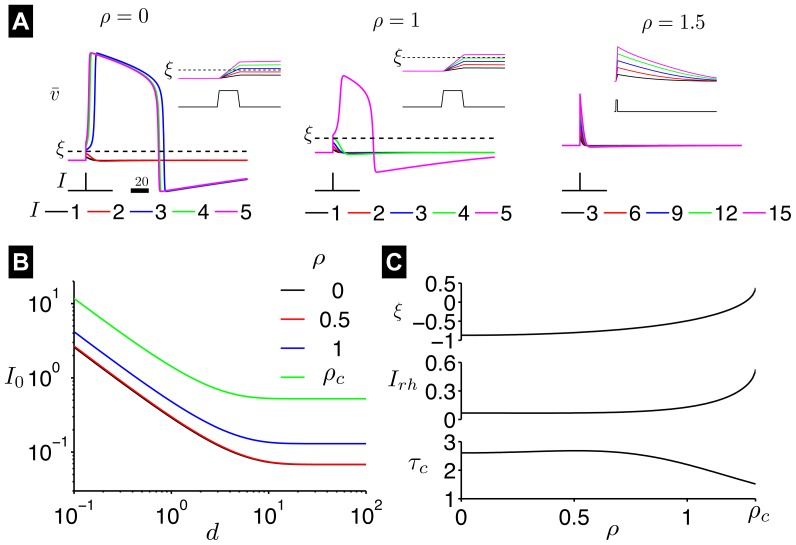
Excitability in the AFHN model. (A) Simulated 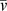 traces during brief 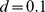 stimuli pulses of amplitude 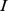 for 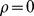, 1, and 1.5. In simulations that 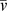 exceeds the threshold 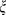, an action potential is elicited. Inset shows an expanded time course. (B) Strength-duration curve ( Eq. 8 ) for several values of 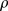. (C) Rheobase (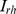, Eq. 9 ) and chronaxie (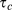, Eq. 10 ) as functions of 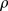.

With the system initial at rest, i.e., 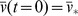, we make the assumption that an action potential is fired when 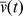 reaches some threshold 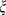. Although it has been shown that the FHN model does not strictly exhibit all-or-none threshold behavior [23], when 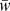 is sufficiently slow compared with 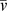, the middle root of the 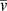-nullcline is a reasonable approximation for an action potential threshold, which we show increases as 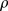 increases (see Methods for details and references on firing threshold, Eq. 44, Figure 4C). This threshold-like behavior is illustrated in Figure 4. We plot 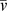 as a function of time following brief 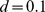 current pulses for 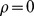 and 1. For both values of 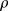, an action potential is elicited if 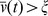 during the brief pulse, while if 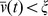 during the current pulse, 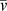 returns to rest 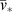. Increasing 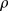 increases both the 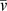 threshold for evoking an action potential, 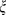, and the stimulus threshold 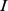 necessary to elicit an action potential (Figure 4C).

In the Methods section, we show a critical value for 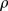, 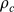, exists, which for all values of 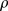,
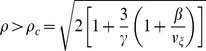
(7)


the AFHN model cannot be excited by a brief applied current, where 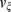 is defined in Eq. 27. Using the parameters used in this study, 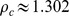. For 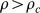, regardless of the magnitude of the stimulus pulse 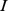, 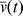 relaxes back towards the steady-state value 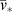 following the applied current pulse, without a large amplitude excursion typical of an action potential (Figure 4A, right panel).

For 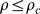, and the strength-duration curve is approximated by
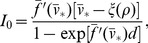
(8)


where the prime indicates differentiation with respect to 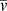, such that
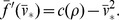



We plot the strength-duration curves in Figure 4B for several values of 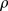. For all values of 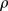, 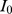 decreases linearly with 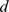 when presented on a logarithmic scale and approaches a constant value for long *d*, a relationship typical of excitable cells. For a given stimulus duration *d*, the strength required to elicit an action potential 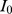 increases as 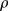 increases. Two important values are typically determined from the strength-duration curves: rheobase (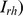, defined as 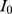 for an infinite duration pulse, and chronaxie (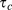), defined as the pulse duration having a threshold that is twice the rheobase. From Eq. 8, 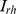 and 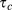 are given by
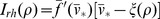
(9)


and
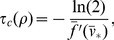
(10)


respectively. We plot 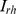 and 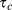 as a function of 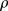 in Figure 4C. Both 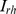 and 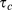 are fairly constant for small 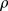. 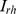 increases and 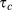 decreases, as 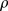 further increases towards 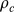. We also plot 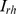 in Figure 2C for comparison with 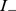, and note that for all values of 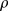, 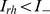, that is, a smaller 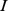 is required to elicit a single action potential than to elicit repetitive spiking, as expected. We note that the derivation of Eq. 8 assumes the stimulus 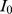 is brief—that is, Eq. 8 is strictly valid for small *d*—therefore, 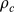 should not be interpreted as a critical 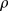 above which no action potentials can be elicited by longer duration stimuli. Indeed, 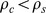, and therefore, the cell can repetitively fire during long duration stimuli for 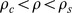, and a single action potential can be elicited by long duration stimuli for 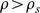. Further, since rheobase is defined as a stimulus threshold for infinite *d*, Eqs. 9 and 10 should be interpreted as approximations derived from Eq. 8, which nonetheless provide qualitative relationships between the strength-duration curve parameters 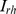 and 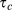 and HF stimulation parameter 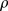 that can be compared with a biophysically-detailed model, as discussed in the next section.

In summary, increasing the HF stimulation parameter 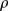 increases the thresholds for both repetitive firing and a single action potential, 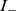 and 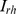, respectively. We derive expressions for critical values of 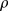 and determine the influence of HF stimulation on the resting potential, firing frequency and amplitude, action potential threshold, rheobase, and chronaxie. These theoretical relationships provide references that can be compared to results from a more realistic neuron model described in the next section.

### Averaged Hodgkin-Huxley model

We next derive and analyze the influence of HF stimulation on a biophysically-detailed model of the neuron, utilizing the techniques described in the previous section. We consider the classical space-clamped Hodgkin-Huxley (HH) neuron model of the giant squid axon [21], with the addition of an applied current 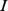 and HF stimulus, given by the following system of equations:
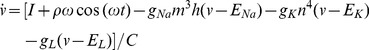
(11a)

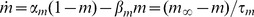
(11b)

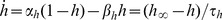
(11c)

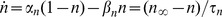
(11d)


where HF stimulus parameters 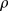 and 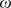 are defined as before. In the HH neuron model, 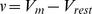 represents the transmembrane voltage 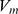 relative to the resting potential 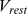, 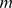 the sodium activation gating variable, 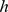 the sodium inactivation gating variable, and 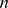 the potassium activation variable. Current conductances, reversal potentials, and gating variable dynamics are described in the Methods.

Assuming that the period of the fast HF stimulation is much shorter than the characteristic times of the dynamics of 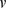 and the gating variables, as in the previous section, we approximate the dynamics of the slow variables by averaging over the period of the HF stimulus. The variables of the averaged Hodgkin-Huxley (AHH) model, 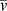, 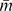, 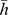, and 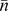, are governed by the following system of equations:
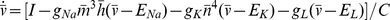
(12a)

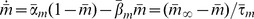
(12b)

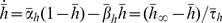
(12c)

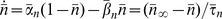
(12d)


where
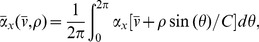
(12e)

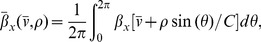
(12f)

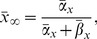
(12g)


and
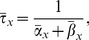
(12h)


for 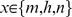. Because of the simplicity of the FHN model, we could derive analytical expressions for the dynamics of the AFHN model variables. In the HH model, the expressions for the 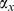 and 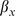 terms that govern the dynamics of the gating variables are complex, and as such, it is not possible to derive analytical expressions for Eqs. 12e and 12f without using approximations for the exponential function. Therefore, Eqs. 12e and 12f are computed by numerical integration for particular values of 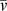 and 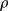.

**Figure 5:**
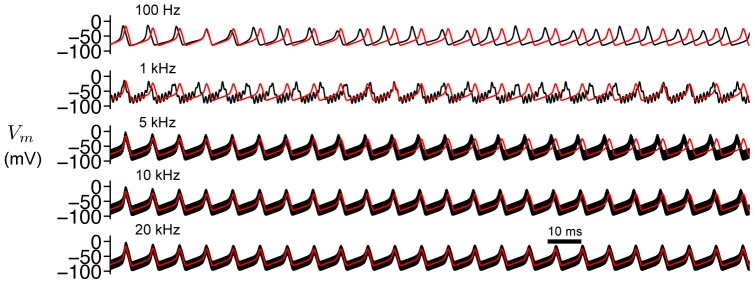
Validation of the AHH model. Simulated 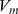 traces from the HH (black) and AHH (red) models for varying HF stimulus frequency 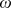. Parameters: 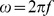 rad/s (where 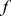 is the frequency identified in each panel), 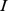  =  30 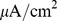, 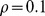 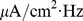.

As with the FHN model, we plot 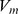 from simulations of the HH model (black) for various values of the HF stimulation frequency and compare with 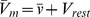 from a simulation of the AHH neuron model (red), for fixed values for 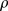 and 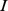 (Figure 5). Below an HF stimulus frequency 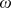 of 5 kHz, there is significant disagreement between the averaged and original model. As 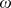 increases, 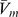 from the AHH model becomes a better approximation of the average value of 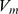 from the HH model simulation, validating the use of the averaging method.

**Figure 6:**
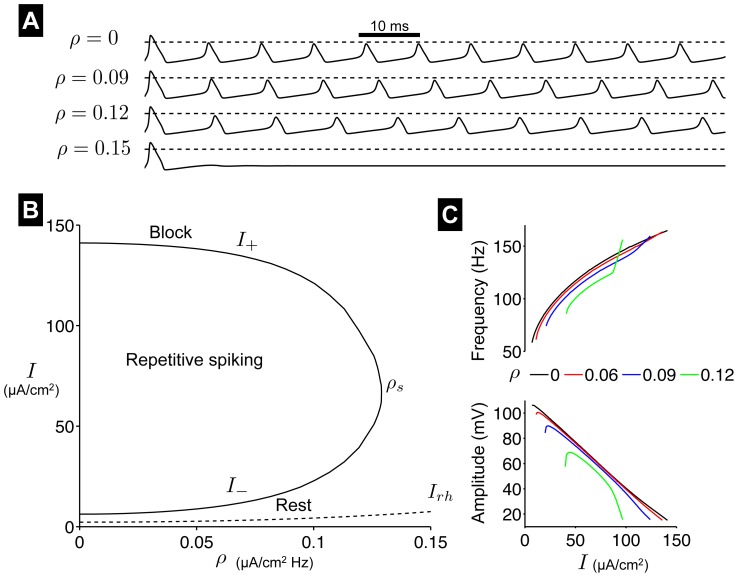
Repetitive firing in the AHH model. (A) Simulated 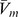 traces for 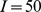 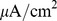 and different values for 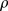. The dashed lines indicate 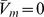 mV. (B) 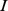-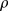 parameter space, denoting regions of rest, repetitive firing, and block. The limit cycle lower and upper limits (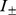) and rheobase (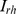) as functions of 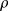. (C) Action potential frequency and amplitude, as functions of 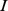 and 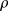. 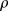 in units of 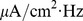.

**Repetitive firing in the AHH model**. As in the previous section, we consider the influence of an applied current 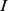 in the AHH model, in the presence of HF stimulation. In Figure 6A, we plot 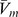 for different values of 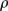, such that the neuron is repetitively firing, i.e., 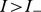. For sufficiently large 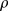, the neuron does not repetitively fire.

We plot the 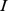-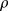 parameter space for the AHH model in Figure 6B. The parameter space is qualitatively similar to the AFHN model, such that the range of 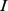 for which the neuron repetitively fires becomes smaller as 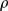 increases, and above a critical value of 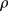, 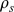, the neuron does not repetitively fire. In the HH model, it has been shown that action potential frequency increases and the action potential amplitude decreases for increasing 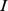 [23], and we find that this is true for a given value of 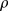. For a given 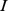, as 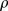 increases, in agreement with the AFHN model, action potential amplitude decreases. However, in contrast with the AFHN model, the frequency decreases as 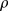 increases (Figure 6C).

**Figure 7:**
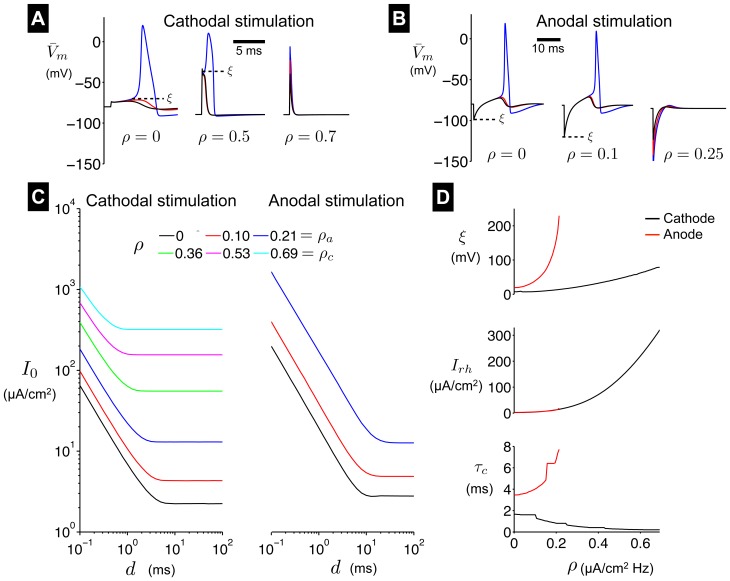
Excitability in the AHH model. (A) 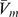 traces following brief (0.1 ms) cathodal and (B) anodal stimulus pulses, for different values of 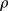. Threshold 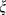 indicated in each panel. (C) Cathodal and anodal strength-duration curves for different values of 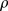. (D) Cathodal and anodal threshold 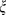 (for 0.1 ms stimuli), rheobase, and chronaxie, as functions of 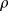. Current pulse amplitudes in (A): 64-66 (left); 633-635 (middle); 600, 800, 1000 (right); in (B): 198-200 (left); 397-399 (middle); 400, 600, 800 (right); in 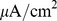.

**Excitability in the AHH model**. We next consider excitability in the AHH model following brief applied current pulses. Here, we consider both positive (cathodal) and negative (anodal) applied current stimuli. As with the FHN model, the HH model is known to not exhibit a strict all-or-none firing threshold. However, especially for brief (0.1 ms) pulses, the HH model demonstrates a threshold-like response. In Figure 7A, we plot 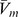 for different values of 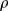 and 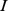. Consistent with the AFHN model, the 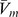 threshold for evoking an action potential, 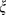, increases for increasing 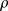 (left, middle panels). Further, above a critical value of 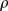, 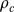, an action potential cannot be evoked, regardless of 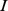 (right panel). Although 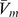 reaches levels near 0 mV, these responses should not be considered action potentials, as the depolarization of 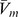 does not arise as a consequence of the regenerative activation of inward currents but rather solely as a perturbation due to the large applied stimulus. Specifically, above 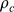, regardless of stimulus amplitude 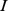, 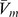 is maximally depolarized as the end of the stimulus pulse and does not become further depolarized following the pulse.

We also consider the influence of HF stimulation on excitability following anodal break stimulation, also known as *post-inhibitory rebound*. In the classical HH model (i.e., 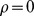), a negative (anodal) applied current pulse 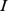 hyperpolarizes the steady-state resting transmembrane potential 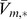 (Figure 7B), which permits sodium inactivation recovery, i.e., 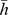 moves closer to 1. Following the pulse offset (break), 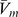 returns towards the more depolarized initial resting potential, and due to the slower sodium inactivation kinetics, 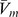 rebound can be sufficiently large to evoke an action potential. As with cathodal stimulation, the threshold for stimulation, 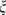 (determined as the magnitude of the hyperpolarization necessary for a post-inhibitary rebound), increases for increasing 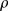 (left, middle panels), and above a critical value of 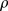, 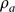 an action potential cannot be evoked (right panel). 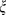 is larger for anodal stimulation (Figure 7D top panel, red), compared with cathodal stimulation (black), and the difference increases as 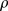 increases, meaning a relatively larger anodal stimulation is necessary to evoke an action potential. Consistent with this finding, we find that 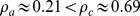 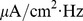.

Strength-duration curves for cathodal and anodal stimulation in the AHH model are shown in Figure 7C. Consistent with the AFHN model, for a given duration, the necessary cathodal applied current strength 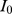 increases as 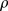 increases. Further, rheobase 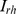 increases and chronaxie 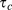 decreases as 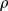 increases, as in the AFHN model (Figure 7D, black traces, middle and bottom panels). As 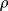 approaches 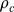, the strength-duration curve becomes flatter, consistent with a decreasing chronaxie, and illustrating that for large 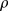 the magnitude of the applied pulse, and not the duration, determine whether an action potential is evoked. For a given 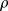, anodal rheobase is slightly larger compared with cathodal rheobase (Figure 7C, D). As 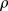 approaches 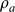, in contrast with cathodal strength-duration curves, the anodal curves become steeper, such that chronaxie increases as 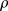 increases (Figure 7D, bottom panel), illustrating that short duration anodal pulses become relatively less effective for evoking post-inhibitory rebound action potentials.

**Dynamics of the AHH model**. For the AFHN model, we demonstrate that 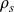 and 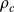 can be approximated via theoretical analysis of the two-dimensional dynamical system, based primarily on analysis of the influence of 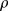 on the phase plane. Various approaches have been used to simplify the HH model to a FHN-like two-dimensional system, often assuming fast sodium activation and a linear relationship between gating variables 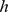 and 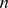 for a given 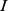 [20]. However, we found that a similar phase plane analysis using this type of reduction of the AHH model was only moderately successful at reproducing AHH dynamics, likely due to the complex relationship between the gating variables dynamics over a wide range of 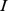 and 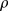 (not shown).

**Figure 8:**
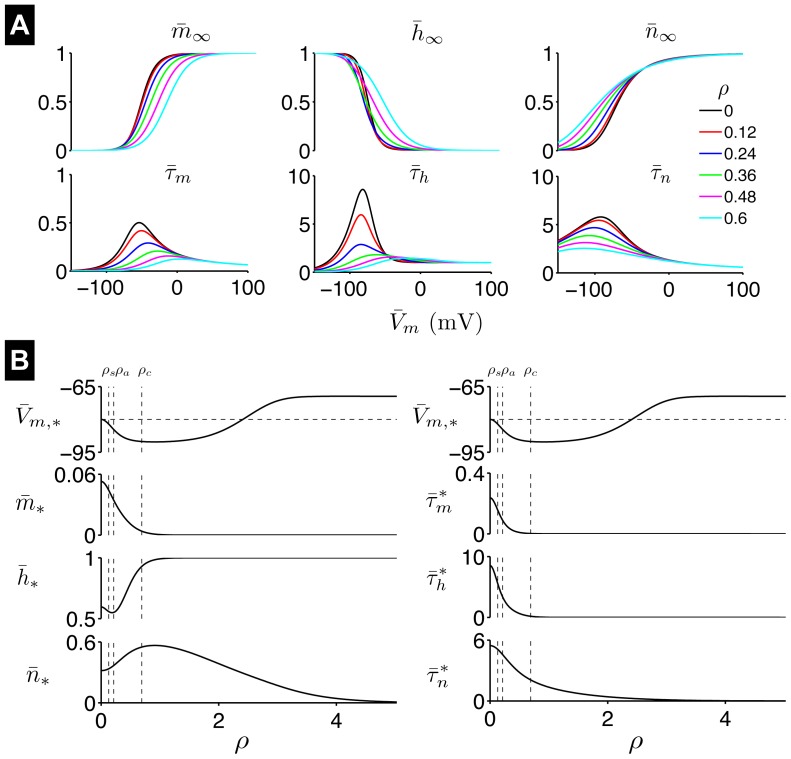
Steady-state of the AHH model. (A) Steady-state gating variables 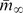, 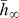, 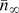 and time constants 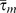, 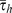, 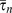 as functions of 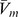 in the AHH neuron model for different 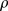 values. (B) Steady-state values for the transmembrane potential 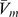 and the gating variables (left), and gating variable time constants at 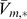 (right), as functions of 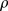. Vertical dashed lines indicate 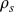, 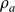, and 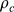 (see text for description). In the top panels, the horizontal dashed line indicates 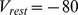 mV for 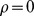. Time constants in units of ms, and 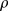 in units of 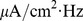.

In the AFHN model, the HF stimulation parameter 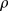 influences the dynamics of 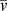 through the cubic function 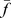. In contrast, in the AHH model the dynamics of 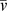 are altered indirectly through the influence of 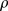 on the gating variables. In Figure 8A, we plot the steady-state activation, inactivation, and time constant curves as functions of 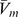 for different values of 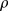. As 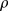 increases, the sodium activation 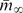 and inactivation 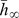 steady-state curves are shifted to the right, the potassium activation 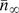 steady-state curve is shifted to the left, and all three curves are less steep (Figure 8A). The time constants 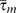, 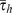, and 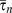 all decrease as 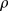 increases.

Shifts in the activation, inactivation, and time constant curves alter the AHH system steady-state (Figure 8B). As 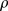 increases, the steady-state transmembrane potential 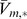 becomes more hyperpolarized, reaching a minimum of 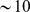 mV *hyperpolarized below* the baseline resting potential 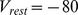 mV. As 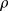 increases further, 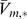 is gradual depolarized, approaching a maximum value of 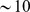 mV *depolarized above* 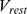. The steady-state sodium activation gate 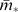 decreases and approaches 0 as 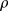 increases. Despite 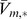 becoming more hyperpolarized for small 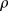, the steady-state sodium inactivation gate 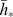 also decreases, and then increases and approaches 1 for large 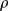. The steady-state potassium activation gate 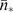 is also complex, first increasing then decreasing and approaching 0 as 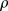 increases.

### Mechanisms of conduction block

The influence of the HF stimulus parameter 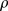 on the dynamics of the gating variables provides significant insight into the mechanism of conduction block in neurons and the various thresholds for repetitive firing and excitability (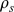, 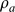, and 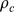) (Figure 8). For small 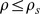 (the critical value of 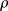 for repetitive firing), the neuron can repetitively fire for some range of the applied current 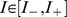. As 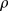 increases, the resting sodium channel activation gate 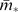 and inactivation gate 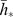 decrease and the resting potassium channel activation gate 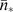 increases, which all drive the neuron towards being less prone to firing.

As 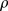 increases for small 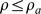 (the critical value of 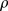 for anodal stimulation), the time constant for sodium channel inactivation 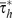 also decreases, that is, following a negative applied current pulse, 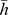 will return to its resting value in a shorter amount of time. Combined with a more hyperpolarized resting transmembrane potential 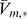 and decreasing 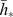, the threshold for anodal excitation 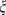 increases dramatically as 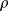 increases (Figure 7). 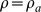 when 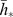 is at a minimum and 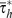 has decreased by approximately a factor of 2.

As 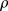 increases for 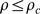 (the critical value of 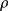 for cathodal stimulation), the sodium activation curve 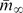 is right shifted, and combined with a more hyperpolarized 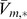 and decreasing 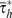, results in an increasing threshold for cathodal simulation 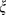 (Figure 7). 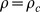 when 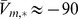 mV and 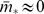 are at a minimum, 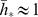 and 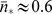 are at a maximum and gating dynamics are fast, that is, the time constants for sodium activation 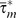 and inactivation 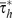 are near 0. Rapid and persistent activation of the potassium current opposes the sodium current and prevents sufficient depolarization to reach the threshold for evoking an action potential. As 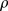 increases for large 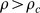, 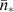 and 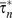 decrease, as 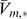 gradually transitions from hyperpolarized to depolarized, relative to 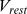. For very large 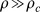, all three time constants are essentially equal to 0, such that the gating variable kinetics can be defined by instantaneous functions of 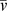. In this regime, the four-dimensional AHH model (Eqs. 12a–12h) is reasonably approximated by a one-dimensional system, for which a large amplitude excursion typical of an action potential is no longer possible. Indeed, for a system with a single stable steady-state (as is the case for large 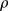), all perturbations from the steady-state are followed by a gradual relaxation back to rest, as observed for large 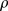 in Figure 7A and B (right panels).

The AHH model suggests that there are different mechanisms of conduction block, depending on the strength of the HF stimulus. For small 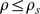, repetitive firing ceases due to decreased sodium channel activation (decreased 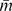) and availability (decreased 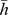) and increased potassium channel activation (increased 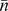). For intermediate 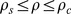, gating variable dynamics are fast (i.e., the time constants approach 0), and therefore eliciting a single action potential via anodal and cathodal excitation fails due to rapid sodium current inactivation, in addition to decreased sodium activation and increased potassium activation. For large 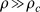, sodium and potassium currents are persistently de-activated (i.e., 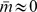 and 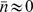), preventing an action potential from being evoked, and 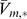 is depolarized due to the influence of the leak current.

In summary, the influence of the HF stimulation parameter 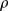 on the properties of action potential firing in the AHH model is similar to that demonstrated in the AFHN model, however with differences in the influence on the resting potential and firing frequency. Further, simulation and analysis of the biophysically-detailed model provides insight into the mechanisms of conduction block. We determined three critical values for 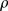, which above the neuron cannot repetitively fire (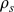), and an action potential cannot be evoked by cathodal (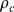) or anodal (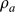) stimulation. Below the critical values, we demonstrated that the thresholds for evoking repetitive firing or a single action potential increase as 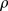 increases.

### HF stimulation of an AHH neuronal network

**Figure 9:**
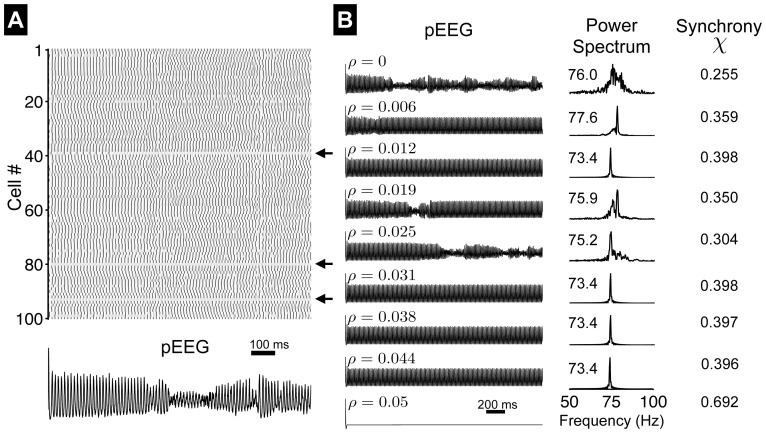
Electrical activity in a network of AHH model neurons. (A) Rastergram of action potentials and the pseudo-electroencephalogram (pEEG). Arrows indicated quiescent neurons. Parameters: 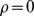, 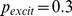. (B) pEEG (left, Eq. 32 ), the corresponding power spectrum (middle, value indicates the average neuron firing rate), and synchrony measure 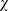 (right, Eq. 33 ), as functions of 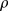. 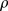 in units of 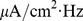.

Finally, we consider the influence of HF stimulation on a random network of 100 neurons, each with, on average, 10 connections, coupled via both excitatory and inhibitory synapses (see Methods for description of synaptic currents and network architecture). Following a single initial applied current pulse, in the absence of HF stimulation (i.e., 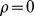), a rastergram shows that most neurons in the network fire repetitively (Figure 9A), while a few neurons remain quiescent due to the absence of incoming excitatory synaptic connections (arrows). The pseudo-electroencephalogram (pEEG, Eq. 32 ) becomes disorganized after an initial time period of synchronization following electrical activity initiation.

We plot the pEEG and the corresponding power spectrum for the same network as 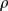 increases (Figure 9B). In general, the average neuron firing rate tends to decrease as 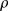 increase, although though this trend is not strictly monotonic with 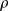 (Figure 9B, middle panels). In general, decreased firing rate is associated with increased network synchronization (Figure 9B, right panels), illustrated by the narrowing of the dominant peak in the power spectrum and increase in the synchrony measure 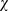 ( Eq. 33 ). When 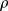 increases further above a critical value of 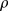, 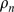, network electrical activity ceases immediately following the initial initiation (Figure 9B, bottom panel, 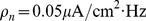 for this example).

**Figure 10:**
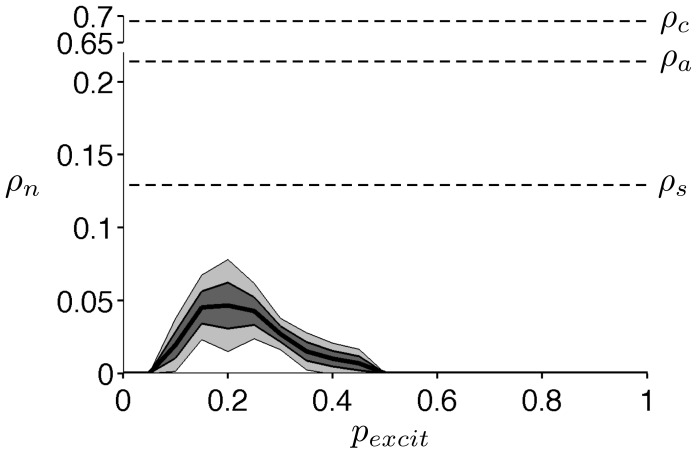
HF stimulation of a network of AHH model neurons. The critical value 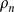 for repetitive activity in a AHH model neural network, as a function of the probability of excitatory synaptic connections 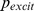. The mean 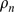 (thick black) over 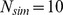 simulations, 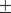 1 (solid black) and 2 (thin black) SEM (standard error of the mean, standard deviation of 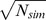) are shown. Single cell critical values of 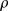 (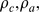 and 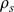) are identified (see text for description). 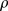 in units of 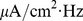.

The critical value 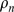 for complete cessation of network electrical activity was highly dependent on the specific architecture of the network and the relative proportion of excitatory and inhibitory synaptic connections (see Methods). We determined 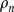 as functions of 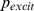, the probability that each synaptic connection was excitatory, and the specific network architecture. For each value of 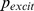, 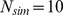 different networks were randomly constructed with the same average connection properties but different connections. The mean value for 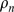, plus/minus one and two standard errors of the mean (SEM, standard deviation divided by 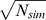) are shown as functions of 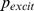 (Figure 10). For all network architectures, 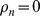 for all networks with 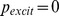 or 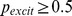, that is, the network does not exhibit persistent activity, even in the absence of HF stimulation. In this specific type of network, persistent network activity requires both excitatory and inhibitory synaptic connections, and indeed that a majority of synapses are inhibitory. For 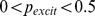, 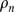 is an inverted U-shaped function of 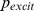, with a maximum near 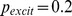. In all networks, the values of 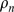 are less than the single cell critical values for repetitive spiking 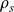, and cathodal and anodal stimulation, 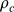 and 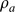, respectively.

## Discussion

### Summary of main findings

In summary, we find that HF stimulation alters the dynamics of excitable cells and networks, resulting in conduction block and electrical quiescence. In a simplified excitable cell model, we identify analytical expressions for HF stimulation strength critical values for repetitive firing and evoking action potentials. In a biophysically-detailed neuronal model, we demonstrate that HF stimulation alters the dynamics of ionic current gating, leading to reduced cellular excitability and conduction block. HF stimulation of a neural network reduces the overall network activity and increases network synchronization, leading to network quiescence for a sufficiently large HF stimulus.

### Relation to prior work

Previous studies have investigated the mechanisms underlying conduction block in neurons [8]–[14]. In these studies, two primary mechanisms for conduction block are posed: persistent potassium current activation of potassium opposing sodium current and preventing the neuron transmembrane potential from reaching a threshold; and reduced sodium channel availability due to a baseline depolarization of the transmembrane potential. It is noted in several studies that these mechanisms are not mutually exclusive and indeed both likely play a role, depending on the species and specific cell type. Our findings are generally consistent with these previous studies, in that we find persistent potassium current activation and reduced sodium channel availability (or de-inactivation). However, a multi-scale method approach permits us to identify how changes in ionic current gating result in different critical thresholds. Further, we are able identify the influence of HF stimulation on the gating variable kinetics, i.e. the gating variable steady-state activation, inactivation and time constant curves, as a significant and novel factor regulating conduction block.

The method of averaging has been previously used to investigate the influence of HF stimulation in the FHN model [17]–[19]. Cubero and colleagues previously demonstrated that HF stimulation can lead to cessation of repetitive firing above a critical threshold (a finding reproduced in the present study) [17]. Ratas and Pyragas determined conditions for which HF stimulation results in slowed and failed propagation. [18], [19]. Here, we extend the approach of these prior studies to demonstrate the influence of HF stimulation on the threshold for evoking a single action potential and additionally to investigate HF stimulation in a biophysically-detailed neuronal single-cell model and network. The FHN model is ideal for mechanistic studies, as the two-dimensional model enables phase-plane analysis and often permits analytical expressions for critical values. We demonstrate that many aspects of HF stimulation of FHN model neurons are similarly reproduced in the HH model, specifically qualitatively similar 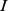-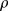 parameter space for repetitive firing; influence of HF stimulation on the action potential threshold; and the existence of critical HF stimulation amplitudes above which neurons cannot repetitively firing or trigger a single action potential.

However, some important properties of HF stimulation of the AHH model are not qualitatively reproduced by the AFHN model, which paints a simpler picture for the mechanism of conduction block. In the AFHN model, the resting potential is gradually depolarized and the gating variable 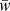 gradually increases as 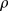 increases, suggesting that conduction block occurs to do the collective influence of transmembrane potential depolarization and a larger degree of refractoriness. In the AHH model, the resting potential is hyperpolarized for small 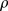 and depolarized as 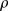 increases. Additionally, the gating variable steady-state values and time constants are altered in a complex manner, such that conduction block is due to both altered refractoriness and the time-dependent dynamics of the refractory variables. The AFHN model also not does reproduce the influence of HF stimulation on firing frequency. In the AFHN model, the frequency increases as 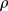 increases. However, in the AHH model, frequency decreases, likely due to the reduced sodium channel availability, i.e. the decrease in 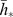, that occurs as 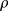 increases for small 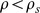. Understanding how HF stimulation influences firing frequency is significant and necessary for optimizing HF stimulation therapy, as frequency plays a significant role in neural computing [27].

### Physiological significance of findings

The term, *high frequency stimulation*, is often used in different clinical settings with different meanings. HF stimulation frequencies range several orders of magnitude from 100 Hz—typical of studies of deep brain stimulation to treat movement disorders such as Parkinson's disease and other neurological disorders such as epilepsy [28], [29]—to 40 kHz—including clinical applications such as pain mitigation and improved bladder voiding [8]–[10]. An inherent assumption in the derivation of the averaged excitable cell models is that the time scale of the HF stimulus is significantly shorter than the time scale of cellular dynamics. We show that the averaged and original HH model begin to agree when the HF stimulation frequency is near 5 kHz (Figure 5), consistent with 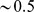 ms (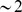 kHz) time-scale for sodium channel activation, and there is greater agreement as the HF frequency increases. This suggests that the method of averaging approach may not be strictly appropriate to the investigation of deep brain stimulation using lower frequency HF in the 100–200 Hz range but highly relevant to the study of peripheral nerve stimulation and clinical applications typically utilizing kilohertz-range HF stimulation. Recently, Weinberg and colleagues demonstrated that HF stimulation in the 100–200 Hz range could block electrical conduction in cardiac tissue, a novel approach to terminate arrhythmias [30], [31]. Since the time scale of cardiac dynamics is generally slower than neuronal dynamics, future work is necessary to determine the validity of the averaging method for investigation of the influence of HF stimulation in cardiac tissue.

In this study, we found that HF stimulation could prevent persistent network electrical activity at lower HF stimulation amplitudes necessary for single cell quiescence, which suggests network activity may cease due to HF stimulation sufficiently reducing excitability in a subset of neurons that prevents re-excitation throughout the network. We only considered network activity that persists (or ceases) following a single initial applied current at the simulation onset. The network response to a constant, repetitive, or random (Poissonian) applied current, in addition to the HF stimulation, may be significantly different. We additionally only consider sparsely connected random network architectures. In this study, we found that the specific network architecture was highly important in determining the response to HF stimulation, and thus it is reasonable to speculate that the network response in highly connected and/or directed neural networks could be different from our findings. Several studies of models including more detailed network architecture and specific cell types have suggested mechanisms underlying deep brain stimulation treatment using HF stimulation in the 100–200 Hz range. Rubin and Terman demonstrated that HF stimulation of the subthalamic nucleus can regularize globus pallidus firing and eliminate pathological thalamic rhythmicity [32]. Using a systems theoretic approach, Agarwal and Sarma demonstrate that HF deep brain stimulation improves reliability of thalamic relay [33]. As noted above, the averaging method may not be strictly appropriate in this frequency range. However, investigation of more physiological neural network architectures and cell types may suggest alternative deep brain stimulation therapies within a higher frequency (kilohertz) regime or provide insight into the role of specific network components with different responses to HF stimulation, as in the aforementioned studies.

In this study, we investigate a simple network with a random architecture and consider the influence of HF stimulation as function of the relative fraction of excitatory synaptic connections. We illustrate a general approach to study HF stimulation in a large neural network which does not require simulation of the HF stimulation term and thus does not require a prohibitively small simulation time step. Here, we consider HF stimulation in the context of only a few network parameters. However, neural networks can exhibit rich and complex dynamics, and much work has demonstrated that the local network architecture can have significant influence on global behavior, e.g., the small-world phenomenon [34]–[36], and network architecture will likely significantly influence our findings. Additional work is necessary to understand the influence of HF stimulation in the context of networks of varying degrees of connectivity and structure.

The critical values for repetitive firing and evoking action potentials are defined in terms of the HF stimulus frequency-to-amplitude ratio; that is, as the HF stimulus frequency increases, so must the HF stimulus amplitude for the same response. In a therapeutic device, it is ideal to minimize the amplitude of an applied stimulus, to minimize power consumption and mitigate safety issues for both the patient and device. Thus, determining the optimal frequency regime to minimize the amplitude for optimal HF stimulation is an important and practical issue. Future work will consider these complications, which must also include analysis of HF stimulation at frequencies approaching the same time scales as cellular dynamics.

### Limitations

In most clinical settings of interest, HF stimulation is applied in the form of an external electrical field stimulus. In this study, we do not account for the influence of an external electrical field nor account for the spatial extent of the nerve axon. Such levels of details are significant for studies of local neural conduction block [8]–[14], as spatial gradients in the extracellular space create virtual electrodes resulting in non-uniform HF stimulation through the nerve axon [37]. It has been shown in multi-compartment models that somatic and axonal firing can become decoupled during 100–200 Hz HF stimulation, such that somatic quiescence does not necessarily preclude activation in neuronal processes [38]. Simulation studies in one-dimensional nerve axons have shown that as the amplitude of a kilohertz-range HF stimulation is increased, the system can transition from regimes of conduction block to rapid firing several times, such that a strict conduction block threshold is not clearly defined [39]. As such, non-uniform stimulation could lead to conduction block in one region of a neuron and rapid activation in another. Further studies of kilohertz-range HF stimulation in more spatially-detailed neuronal models are necessary to investigate these complex issues.

The HH model of the giant squid nerve axon is a classical model of an excitable cell, highly studied and well-characterized, and thus it was a reasonable biophysically-detailed ionic model to characterize the influence of HF stimulation using the method of averaging approach. However, several more detailed neuronal models relevant to mammalian physiology have been described, incorporating more detailed and multiple sodium, potassium, and calcium currents [40]–[42]. Indeed, the interaction between voltage-gated calcium channels and calcium-mediated synaptic transmission may be important for understanding the influence of HF stimulation on network activity and designing an optimal therapy and warrants further study.

Finally, the simulation results presented here are deterministic and do not account for stochastic fluctuations inherent in neuronal signaling at both the cellular and subcellular levels. Indeed, studies have shown that noise-induced firing can be enhanced by HF stimulation for sufficiently large noise levels, termed vibrational resonance [17], closely related to the well-known phenomenon of stochastic resonance [43]. Further work is necessary to investigate the influence of stochastic fluctuations on spiking in biophysically-detailed averaged neuronal models.

## Methods

### Derivation of AFHN model

We derive the AFHN model equations, following the approach in [19]. See [18], [19] for a more details. For HF stimuli with large frequencies 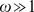, the period of HF oscillations is much less than the characteristic time scales of the FHN neuron. Therefore, we seek to eliminate the HF stimulus term 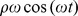 from Eq. 1a and obtain an autonomous system which approximates the original system on the time scale of the FHN neuron. First, we change the variables in Eqs. 1a and 1b, substituting
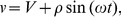
(13a)

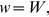
(13b)


and derive the following equations for 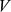 and 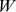:
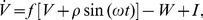
(14a)




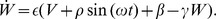



Mathematically, we are interested in the limit 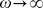, for a fixed 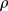. By rescaling time 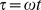, we can transform the system to
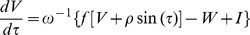
(15a)

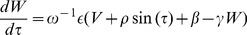
(15b)


The variables 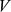 and 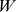 vary slowly relative to the periodic function 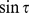, due to the small parameter 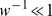. According to the method of averaging [15], an approximate solution to the system can be obtained by averaging over the fast periodic function, and the averaged variables 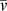 and 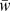 satisfy the following ODEs:
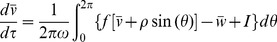
(16a)

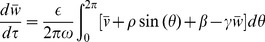
(16b)


After calculating the integrals and returning to the original time scale, the averaged system is as given above in Eqs. 2a and 2b. Importantly, we note that the assumption implicit in stating Eq. 13a, specifically that the original system voltage 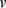 can be expressed as the sum of slow varying 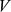 and high frequency term 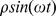, underlies an important conclusion of the method of averaging theorem [44]: an equilibrium point in the averaged system (e.g., rest or depolarization block) corresponds to a periodic solution in the original system (due to the additional HF term superimposed on top of the averaged system equilibrium). Similarly, a periodic orbit in the averaged system (e.g., repetitive firing) corresponds to a more complex oscillation or tori, observed in the 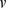 traces of the FHN and AFHN models in Figure 1.

### Firing frequency and amplitude in the AFHN model

**Figure 11:**
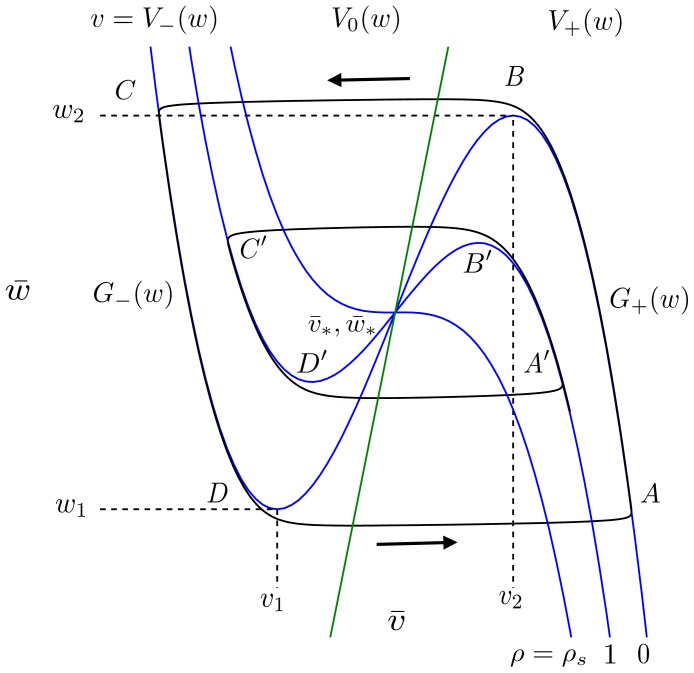
Period of a stable limit cycle in the AFHN model. For 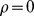, the period 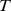 of the stable limit cycle is approximately the sum of the time required to traverse the two stable branches of the 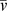-nullcline, 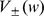, denoted by the points 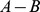 and 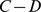, respectively. As 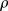 increases, the amplitude and period of the stable limit cycle, indicated by the second set of points labeled 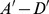, both decrease. See text for description of other variables in figure. Parameters: 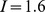.

Following a similar approach as described in [24], expressions for the firing frequency and action potential amplitude in the AFHN model are derived as follows. Recall from Eqs. 2a and 2b that the 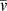-nullcline 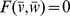 is cubic in shape. Therefore, over the finite range of values for 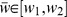, there are three solutions of the equation 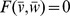, which we can denote by 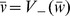, 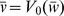, and 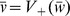 (see Figure 11). The minimal value of 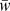 for which 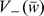 exists is 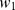, the maximal value of 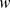 for which 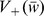 exists is 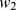, both of which are functions of 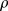 and 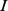. 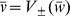, the left and right branches, are termed the stable branches of the 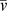-nullcline, and 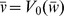, the middle branch, is termed the unstable branch, because, in the limit that 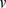 is much faster than 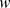, a steady-state located on the (un)stable branch is (un)stable.

The locations of (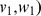 and 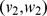 are given by the local minimum and maximum, respectively, of the 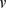-nullcline, given by
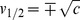



and
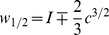



Because 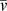 is fast compared to 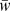, 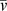 rapidly moves between stable branches of the 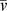-nullcline, 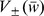. We can approximate the period of an oscillation 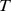 by the time required to travel along the two stable branches 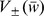


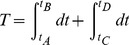
(17)


where points 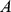-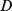 are indiciated in Figure 11. Along the stable branches, the dynamics of 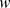 are determined by
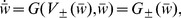
(18)


and, therefore, Eq. 17 is equivalently given by
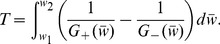
(19)


Note that both terms of the integral are positive, since 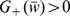 and 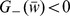 over the range of values 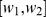. The frequency of oscillations is given by 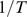.

From Figure 11, we observe that the amplitude of the limit cycle—and thus, of the action potential—is given by 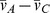, the difference between the values of 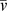 at points 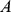 and 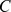, respectively. 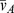 is the non-repeated root solution of
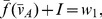
(20)


and 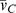 is similarly the non-repeated root solution of
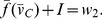
(21)


### Strength-duration curve in the AFHN model

An approximation for the strength-duration for the AFHN model is derived as follows. Near the steady-state 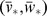, the dynamics of 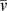 can be approximated by
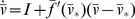
(22)


where the dot indicates differentiation with respect to time and the prime indicates differentiation with respect to 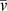, such that
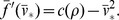



and 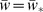. Solving Eq. 22 with the initial condition 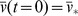,
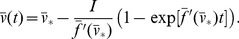
(23)


We set 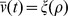, the 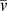 threshold for eliciting an action potential, and after rearranging, we arrive at the strength-duration curve relationship in Eq. 8.

**Figure 12:**
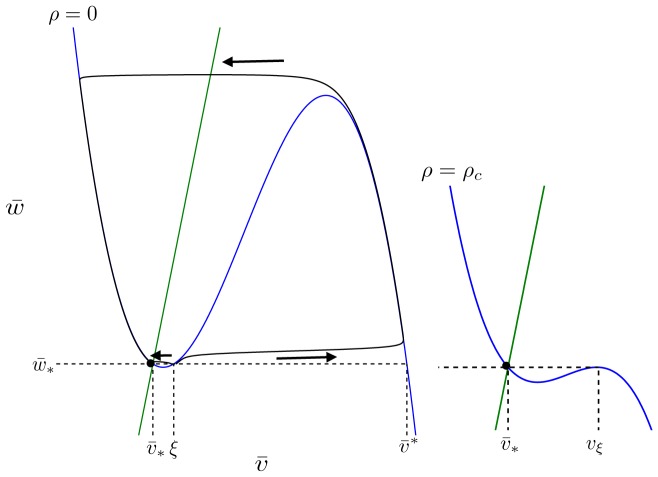
Sub- and super-threshold brief stimuli in the AFHN model. (Left) For 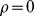, 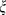 indicates the threshold for evoking an action potential. Two trajectories starting near 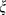 are identified by arrows: (1, left arrow) when the initial condition 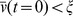, 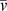 returns quickly to the resting potential 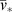; (2, right arrow) when 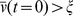, an action potential is evoked: the system follows a counterclockwise trajectory, quickly approaching the 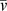-nullcline, following the right stable branch until reaching the right knee, quickly reaching the left stable branch, and then returning to rest. (Right) When 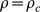, 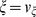, the critical value above which an action potential cannot be evoked by a brief perturbation from the steady-state.

To evaluate Eq. 8, we must determine the dependence of the threshold 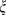 on 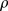. Many studies have discussed the absence of a well-defined threshold in the FHN model [45]–[47]. Izhikevich notes that canard trajectories following the repelling slow manifold provide the best approximation to the excitability threshold [45]. Recent work has show that this manifold is well approximated by inflection sets (regions of flow lines with zero curvature in the phase plane) [46]. For simplicity, we approximate the threshold 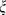 by the middle solution of
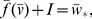
(24)


which, as shown in Figure 12 (left panel), does reasonably well-approximate the action potential threshold. We are interested in the threshold 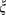 from a non-stimulated state, i.e., 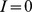. Therefore, Eq. 41 can be written as
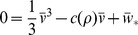
(25a)

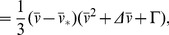
(25b)


where Eq. 25b is implied, since 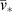 by construction is a solution of Eq. 25a. Matching coefficients, 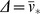 and 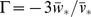, and using the quadratic formula, 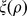 is given by
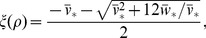
(26)


where the dependence of 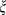 on 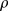 is due to the dependence of 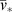 and 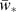 on 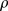. Further, from the definition of 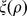, if the terms under the radical equal 0, 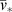 is equal to a critical value, which we will call 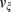
—above which the threshold is ill-defined, that is, the system cannot be excited by a brief perturbation from the steady-state. Using Eq. 3b, 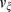 is the real solution of the cubic equation
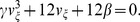
(27)


Note that 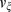 does not depend on 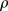, only the system parameters. Using Eqs. 25a and 27, for all values of 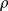, if
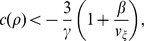



and therefore,
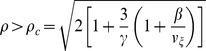
(28)


the system cannot be excited by a brief stimulus pulse, illustrated in Figure 12 (right panel).

### Hodgkin-Huxley model equations

The equations governing the dynamics of the gating variables 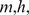 and 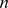 are given by
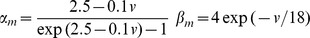


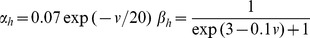


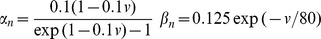



where 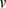 is the shifted transmembrane potential, in which the resting potential 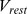 has been subtracted. The standard parameters for the HH model are given in Table 1.

**Table 1:** Hodgkin-Huxley model current parameters.

Parameter	Definition	Units	Value
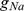	maximum Na 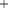 current conductance	mS/cm 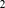	120
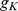	maximum K 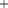 current conductance	mS/cm 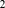	36
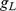	maximum leak current conductance	mS/cm 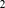	0.3
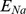	Na 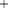 current reversal potential	mV	115
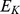	K 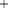 current reversal potential	mV	–12
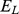	leak current reversal potential	mV	10.6
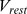	resting potential	mV	–80
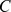	capacitance	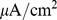	1

Parameters for ionic currents in the HH model.

### Neuronal network model and architecture

A network of 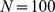 AHH neurons are simulated by adding a synaptic current to the AHH model, such that the 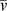 dynamics of the 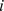-th neuron are governed by the following equation:
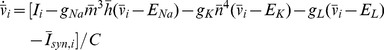
(29)


where
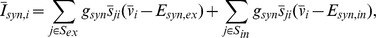



and 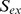 and 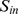 are the set of presynaptic neurons with connections to neuron 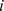, with excitatory and inhibitory, respectively, synapses. 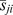 is the averaged gating variable for the postsynaptic conductance, and assumed to be an instantaneous, sigmoidal function of the presynaptic cell potential with a threshold 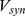 [48], that is
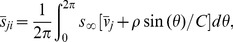
(30)


where
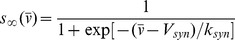
(31)


Parameters are in Table 2. The specific synaptic connections between neurons are determined randomly, as follows. The number of presynaptic connections to the 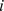-th neuron is drawn from a Gaussian distribution with mean 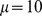 and standard deviation 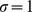, rounded to the nearest whole number. The presynaptic neuron indices 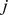 are chosen at random. The type of each synapse, excitatory or inhibitory, is determined at random, such that the probability of an excitatory synapse is 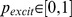. Electrical activity is evoked in the neural network by applying a 200-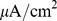, 0.1-ms applied current in 50 randomly selected neurons at time 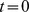.

**Table 2:** Synaptic current parameters.

Parameter	Definition	Units	Value
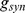	maximum synaptic conductance	mS/cm 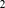	0.3
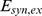	excitatory synapse reversal potential	mV	80
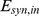	inhibitory synapse reversal potential	mV	–12
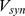	presynaptic cell potential threshold	mV	50
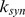	threshold parameter	mV	2

Parameters for excitatory and inhibitory synaptic currents in a network of AHH model neurons.

The collective activity of the neural network can be represented by the pseudo-electroencephalogram (pEEG) [49], given by 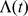,
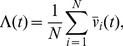
(32)


the transmembrane potential averaged over all neurons. The frequency-domain representation of the pEEG is computed by the Fast Fourier Transform.

The synchrony of the electrical activity in the network is given by the synchrony measure 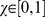 [50],
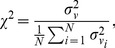
(33)


where the variance of the time fluctuations of the average transmembrane potential, 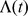,
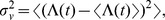



the variance of the time fluctuations of the individual transmembrane potentials 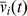,
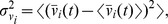



and 
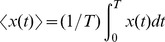
 denotes time-averaging over the duration of the simulation 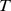. Note that if 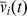 are identical for all 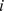, then 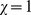.

### Numerical simulations

All numerical simulations were performed in MATLAB. For simulations of the AHH model, the modified gating variable rate functions (Eqs. 12e–12f) were pre-calculated for a given value of 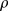 for 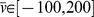 mV (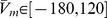 mV), and values were linearly interpolated from look-up tables during simulations.
